# Controlling Magnetization in Ferromagnetic Semiconductors by Current-Induced Spin-Orbit Torque

**DOI:** 10.3390/ma18020271

**Published:** 2025-01-09

**Authors:** Sanghoon Lee, Xinyu Liu, Jacek Furdyna

**Affiliations:** 1 Physics Department, Korea University, Seoul 136-701, Republic of Korea; 2 Department Physics and Astronomy, University of Notre Dame, Notre Dame, IN 46556, USA; xliu2@nd.edu

**Keywords:** ferromagnetic semiconductors, spin-orbit interactions, Rashba effect, Dresselhaus effect, bulk inversion asymmetry, spin-orbit torque, spin polarization, magnetization switching, spin texture

## Abstract

In this paper, we review our work on the manipulation of magnetization in ferromagnetic semiconductors (FMSs) using electric-current-induced spin-orbit torque (SOT). Our review focuses on FMS layers from the (Ga,Mn)As zinc-blende family grown by molecular beam epitaxy. We describe the processes used to obtain spin polarization of the current that is required to achieve SOT, and we briefly discuss methods of specimen preparation and of measuring the state of magnetization. Using specific examples, we then discuss experiments for switching the magnetization in FMS layers with either out-of-plane or in-plane easy axes. We compare the efficiency of SOT manipulation in single-layer FMS structures to that observed in heavy-metal/ferromagnet bilayers that are commonly used in magnetization switching by SOT. We then provide examples of prototype devices made possible by manipulation of magnetization by SOT in FMSs, such as read-write devices. Finally, based on our experimental results, we discuss future directions which need to be explored to achieve practical magnetic memories and related applications based on SOT switching.

## 1. Introduction and Background Information

The magnetization of a ferromagnetic semiconductors (FMSs) holds excellent potential for memory device applications, because the electronic properties of these materials—e.g., of (Ga,Mn)As—can be effectively used to control and manipulate their magnetic properties. Magnetization can of course be manipulated by an external magnetic field. This approach, however, is not suitable for individually switching the magnetization within an array of densely packed devices. However, it is also possible to manipulate magnetization by means of electric current by methods that involve spin-orbit interactions [1]. This approach, in addition to its ability to control magnetization in individual small-scale devices, also has the advantages of fast switching speeds and low energy consumption, thus making it especially suitable for device applications. In this paper, we review our recent work on this topic, applying a wide range of materials and geometries.

### 1.1. Spin Polarization of Electric Current in FM Semiconductors

**Figure 1:**
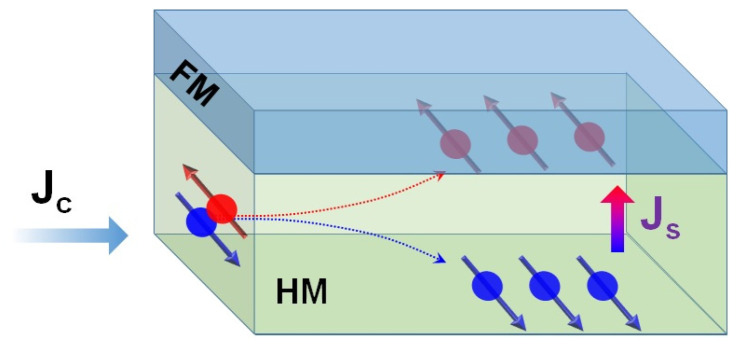
Schematic of spin current transport in HM/FM system. Spins of opposite sign (represented by red and blue dots) in the originally unpolarized current **J_c_** are separated by the spin Hall effect in the HM layer. The spins represented by red dots are immediately adjacent to the FM layer and can thus interact with its magnetization.

The manipulation of magnetization by electric current requires that the current be spin polarized, so that magnetic moments of current carriers can interact with the magnetization of the material. For example, in bilayers comprised of a heavy metal (HM) and a ferromagnet (FM) (see Figure 1), the spin Hall effect in the heavy metal layer separates the spins between two surfaces, and if one of these surfaces is interfaced with the FM layer, the current spin adjacent to that layer can then interact with the its magnetization and can be employed for magnetization switching and other forms of magnetic manipulation [2,3].

Here, ferromagnetic semiconductor layers of the (Ga,Mn)As family offer the important advantage that the electric current in such systems is automatically spin-polarized, owing to inversion asymmetry that is naturally present in these materials. There are two processes whereby an electric current can be spin-polarized in a layer of (Ga,Mn)As-type material. First, the zinc-blende crystal structure of these FM semiconductors has a broken inversion symmetry, which (through relativistic considerations and spin-orbit interactions) leads to the so-called Dresselhaus field [4]. Second, since we are interested in specimens in layer form, the layer structure automatically has a structural inversion asymmetry, which (also through relativistic considerations) results in a spin polarization of the current, as first noted by Rashba and Bychkov [5,6].

**Figure 2:**
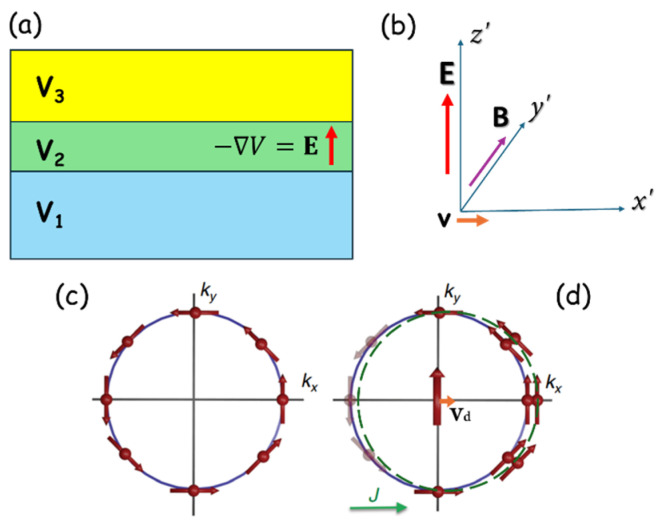
Schematics of (**a**) electric field E generated by the discontinuity of potential V due to structural discontinuity. (**b**) Magnetic field B “felt” by a charge carrier moving with velocity v relative to the laboratory frame: (**c**) Rashba spin-texture of the Fermi sphere; and (**d**) non-equilibrium redistribution of eigenstates on the Fermi sphere for current J in the *x* direction. Here $k_{x}$ and $k_{y}$ are wave vectors and $v_{d}$ is the drift velocity of current carriers.

As an illustration, let us first consider the structural (or macroscopic) inversion asymmetry (SIA) that is characteristic of every layer structure, where the breaking of inversion symmetry is caused by the discontinuity of the material at the interfaces [5,6], as shown in Figure 2. The discontinuity of the potential V due to structural discontinuity will necessarily generate an electric field (E=−∇V) along the direction normal to the layer. Now consider a current flowing in the layer plane, i.e., perpendicular to the field E arising from this structurally induced potential gradient. The current carriers necessarily move in electric field E, as shown in Figure 2; but in their frame of reference, they feel a magnetic field B given by Lorentz transformation $B=-\gamma \left(v\times E\right)/c^{2}$ (where v is the carrier velocity, c is the speed of light, and γ is Lorentz factor), which then orients their spin. The above simple description is a “toy model”, since it does not take core charges into account, but it shows the basic concepts of how a current becomes spin-polarized.

**Figure 3:**
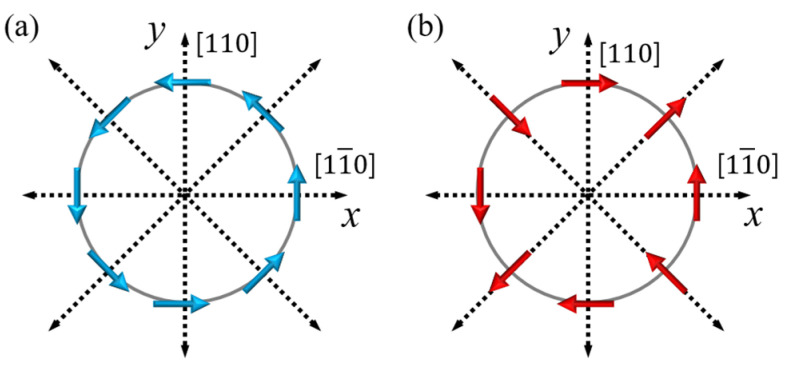
Directions of Rashba (**a**) and Dresselhaus (**b**) fields, shown by blue and red arrows, respectively, for current directions indicated by dotted arrows.

The electric field due to the bulk inversion asymmetry (BIA) of the crystal is more difficult to visualize, since it depends, in a complicated way, on crystallographic directions; we discuss this in the Appendix A. The idea, however, is the same: due to their motion, charge carriers of the current “feel” a magnetic field resulting from the Lorentz transformation, which then polarizes their spin. The full effects of BIA and SIA are contained in the Hamiltonian of the system that includes spin-orbit (SO) interactions, shown below [7,8,9]:

(1) $$H=\frac{\hbar^{2}k^{2}}{2m}+U\left(x\right)+H_{SO}=\frac{\hbar^{2}k^{2}}{2m}+U\left(x\right)+a\left(\sigma_{x}k_{y}-\sigma_{y}k_{x}\right)+b\left(\sigma_{x}k_{y}+\sigma_{y}k_{x}\right),$$

where k is the electron wave vector and σ is the vector of the Pauli matrices in a coordinate system with $x∥\left[1\bar{1}0\right]$, y∥[110], and z normal to the layer. The form of BIA contribution, given by Equation (1), arises from for the zinc blende structure, which may be strained in epitaxially grown layers due to a mismatch between the substrate and the layer of interest. Thus, the BIA contribution, and therefore, constant b in Equation (1), depend strongly on the strain in the system [10,11]. From the above spin Hamiltonian, we can show that the relativistically induced fields due to SIA and BIA are [12]:

(2) $$B_{\mathrm{SIA}}=\frac{\alpha }{g\mu_{B}}\left(\begin{array}{c} k_{y}\\ -k_{x} \end{array}\right),\;\;B_{\mathrm{BIA}}=\frac{\beta }{g\mu_{B}}\left(\begin{array}{c} k_{y}\\ \;k_{x} \end{array}\right),$$

where $\mu_{B}$ is the Bohr magneton, g is the electronic g-factor, and α and β are Rashba and Dresselhaus coupling constants. The relationship of these fields relative to the carrier motion (and thus to the current) is shown in Figure 3.

While Equation (2) and Figure 3 are specifically obtained for zinc blende structures, one should note that formation of both SIA and BIA fields (and thus spin polarization of the current) occurs in all crystal structures with broken inversion symmetries, regardless of whether the system is magnetic (of interest in the present paper) or non-magnetic.

### 1.2. Interaction of Magnetization of FMS with Spin-Polarized Current

Now consider such a spin-polarized current flowing in a ferromagnetic medium, such as (Ga,Mn)As and related materials. The magnetic moment of such a spin-polarized current will then interact with the magnetization M of the material, as described by the Landau-Lifshitz-Gilbert (LLG) equation, where M is the magnetization of the FM medium,

(3) $$\frac{dM}{dt}=-\gamma_{0}\left[M\times H_{eff}\right]-\frac{G}{\gamma_{0}M^{2}}\left[M\times \left(M\times H_{eff}\right)\right]+b\;\sigma \times M+\;a\;M\times \left(\sigma \times M\right),$$

$H_{eff}\;$ is the total magnetic field (i.e., the applied external field and the anisotropy field), $\gamma_{0}$ is the electron gyromagnetic ratio, G is Gilbert damping and σ is the spin polarization of the current. The interaction of the spin-polarized current carriers with M then constitutes the mechanism for manipulating magnetization of the material by the current. In Equation (3), the term b σ×M  is referred to as the field-like torque (FLT), and the term *a* M×(σ×M) as the damping-like torque (DLT), in an analogy with the first two terms of the LLG equation [13,14,15]. We will further recognize that DLT is typically much larger than FLT and is thus the dominant term of the interaction between σ and M [16,17], tending to align M with σ.

### 1.3. Magnetic Anisotropy of Ferromagnetic Semiconductors

**Figure 4:**
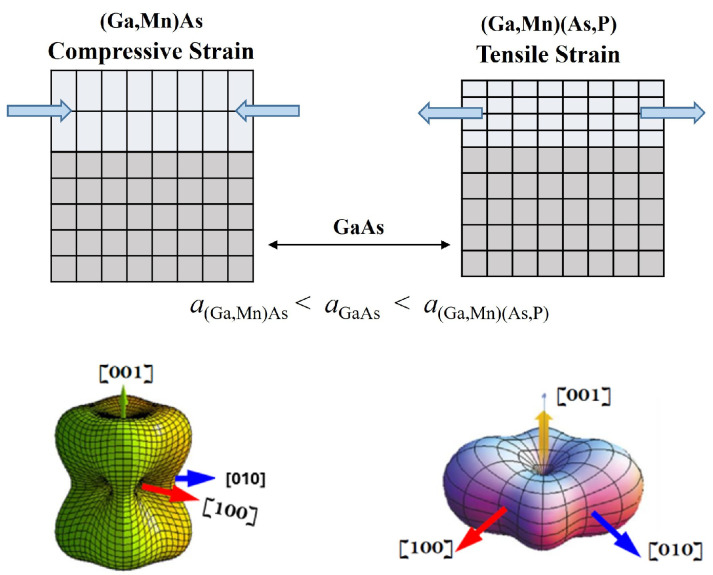
(**Top**) panel: Schematics of strained epitaxy films as the (Ga,Mn)As and (Ga,Mn)(As,P) films adapt the in-plane lattice spacing of the GaAs substrate. (**Bottom**) panel: 3D plots of free energy density at zero field for films with compressive (**left**) and tensile strain (**right**). Arrows indicate crystal directions, where energy minima appear. Parts of the figure are taken, with permission by author, from [18].

In this review, we are interested in ferromagnetic semiconductors such as (Ga,Mn)As, (Ga,Mn)(As,P), (Ga,Mn)(Bi,As), Ref. [18] etc. These are all cubic materials, but when grown on a substrate with different lattice parameters, they will be under tensile or compressive strain due to the lattice constant differences. This then affects the symmetry of their magnetization [19,20,21], as seen in the magnetic free energy diagrams shown in Figure 4.

In the case of a compressively strained (Ga,Mn)As film grown on (001) GaAs substrate, the energy minima appear in the (001) plane near the <100> directions and are strongest along the [100] and [010] direction, indicating that the dominant magnetic easy axes lie in the film plane. When the strain is tensile, as in a (Ga,Mn)(As,P) film grown on a GaAs substrate, the deepest energy minimum occurs along the [001] direction, indicating that in this strain condition, the film anisotropy is dominated by an out-of-plane magnetic easy axis [22].

## 2. Materials Preparation and Experimental Methods

### 2.1. Materials Preparation

All (Ga,Mn)As and (Ga,Mn)(As,P) specimens used in these studies were grown by low-temperature molecular beam epitaxy (MBE) on semi-insulating GaAs (100) substrates. High-purity elemental fluxes of Ga and Mn were supplied by standard effusion cells, while As_2_ and P_2_ fluxes were generated by cracker cells. Growth was monitored in-situ using reflection high-energy electron diffraction (RHEED) [23,24]. During the growth of the (Ga,Mn)As and (Ga,Mn)(As,P) layers, the substrate temperature was maintained at 250 °C. The Mn flux was controlled by adjusting the temperature of the Mn effusion source, while the Ga effusion cell temperature remained constant. The As_2_ flux was also held constant, with a beam equivalent pressure (BEP) ratio of As_2_:Ga of approximately 5. The growth rate of the films (0.2 nm/s) was estimated based on variations in source flux. After the growth, the concentrations of manganese and phosphorus, film thicknesses, Curie temperatures, and saturation magnetizations were determined by high-resolution X-ray diffraction and SQUID magnetometry using a Quantum Design MPMS XL system.

### 2.2. Monitoring Magnetization by Hall Resistance Measurements

The key experiments used in these studies were aimed at determining the magnitude and orientation of magnetization. For that purpose, it was convenient to use Hall resistance (Hall voltage divided by the current), since in this way we determined both the magnitude of magnetization M and its relation to the current direction. Hall resistance in ferromagnets is given by the relation [25]

(4) $$HR=\frac{R_{o}}{t}\;H_{⊥}+\frac{R_{s}}{t}\;M_{⊥}+\frac{k}{t}M_{∥}^{2}\sin2\varphi_{M},$$

where the first term is the ordinary Hall resistance, the second is the anomalous Hall resistance (AHR), and the third is the planar Hall resistance (PHR). Note, however, that in a ferromagnet, the terms depending on M are much larger than the normal Hall term, and in the context of the present paper, we will only rely on the last two terms of Equation (4). Specifically, if M is perpendicular to the layer, we can determine its state by using the AHR term, $HR=\frac{R_{s}}{t}\;M_{⊥}$; and if we are interested in manipulating the in-plane magnetization by the current, we will use the PHR term, $\frac{k}{t}M_{∥}^{2}\sin2\varphi_{M}$.

## 3. Experimental Results and Discussion

### 3.1. Reversal of M Normal to Layer Plane

#### 3.1.1. Mechanism of Magnetization Reversal

**Figure 5:**
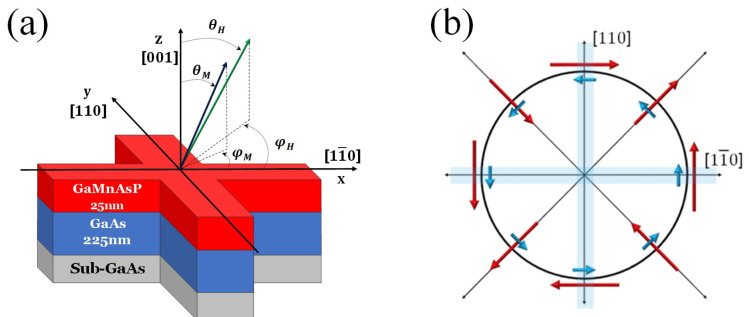
(**a**) Schematic diagram of a crossbar device patterned on Ga_1−*x*_Mn*_x_*As_1−*y*_P*_y_* film grown on GaAs (001) substrate, including the coordinate system used in these studies. (**b**) The directions of the Rashba and Dresselhaus SOI fields are shown by blue and red arrows, respectively, for current directions, which are indicated by thin solid arrows. Note that for the current along $\left[1\bar{1}0\right],\;\mathrm{the}\;\mathrm{two}\;\mathrm{fields}\;\mathrm{are}\;\mathrm{parallel}$; for the current along [110], the fields are anti-parallel.

**Figure 6:**
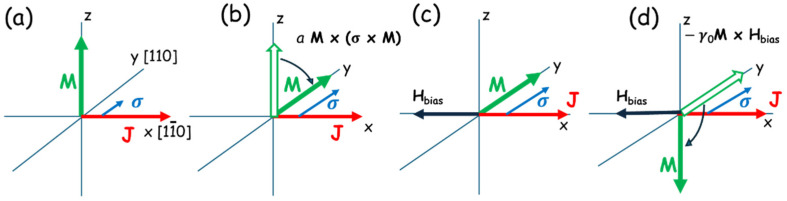
Schematic of SOT switching process for out-of-plane magnetization. (**a**) Magnetization is initialized by an external field, which is then removed. (**b**) As the current J increases, the torque of its spin polarization turns M from its initial orientation (empty green arrow) to the direction of σ (full green arrow). (**c**) $H_{\mathrm{bias}}$ is applied along the −x direction and exerts a torque $-\gamma_{0}M\times H_{\mathrm{bias}}$ on M, turning it below the xy-plane. (**d**) Once this occurs, the anisotropy field turns M to its final orientation along −z. The initial and final direction of M in each panel is shown by open and full greens arrows; the directions of the current, bias field, and spin polarization are shown by red, black, and blue arrows, respectively.

Let us first discuss the manipulation of M when it is perpendicular to the layer plane, the situation that occurs when the easy axis is normal to the layer, as in the case of films of the (Ga,Mn)As family under tensile strain, as shown in Figure 4. To facilitate further discussion of experiments, in Figure 5, we show a typical sample arrangement, where M is aligned in the +z direction, for example by an initiating strong magnetic field that is applied along +z and then removed. According to the LLG equation, when the current through the FMS layer is spin-polarized, the spin of the current σ  will act on M through the damping-like term *a* M×(σ×M) in the LLG equation. But in this case, the spin-polarized current can turn M from its original out-of-plane orientation only to an in-plane orientation, aligning it along σ. To get M below the xy-plane, we need an additional in-plane bias field H—in this case, opposite to J [16,26]. For clarity we show this process by the sequence of separate steps described in Figure 6.

Whether the bias will turn M below or above the xy-plane will depend on the orientation of bias field H. However, once the bias turns M below the xy-plane, the magnetic anisotropy of the system turns it to align with the vertical easy axis, completing the magnetization reversal, as shown in Figure 6.

#### 3.1.2. Example of Magnetization Switching Measurements

**Figure 7:**
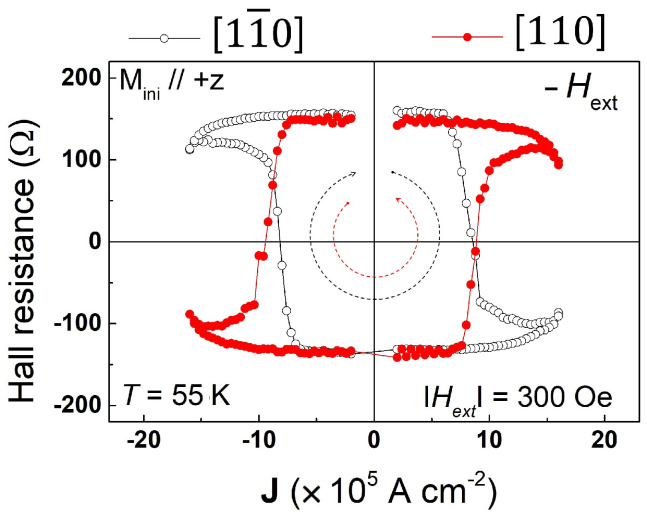
Hall resistance hystereses observed in a (Ga,Mn)(As,P) film at 55 K for currents scanned along $\left[1\bar{1}0\right]$ direction (black dots) and [110] (red dots), indicating current-induced magnetization switching in the film. Switching chiralities of the $\left[1\bar{1}0\right]$ and [110] hystereses are indicated by red and black dashed arrows, respectively. Adapted from Ref. [26].

Numerous studies on reversing perpendicular magnetization by SOT have already been carried out by various research groups on a variety of FMS systems [16,26,27,28]. Let us illustrate the process by experiments carried out on layers of (Ga,Mn)(As,P) on GaAs, where the magnetization is automatically perpendicular to the layer plane owing to tensile strain in the FM layer. In Figure 7, we show AHR hystereses observed using the sample arrangement shown in Figure 5 as the currents are swept either along [110] or $\left[1\bar{1}0\right]$. As an example, consider the results where the initial magnetization is in the +z direction, as shown in Figure 7. When the current flows in the positive $\left[1\bar{1}0\right]$ direction (black open circles), we must apply a bias field $H_{\mathrm{bias}}$ opposite to the current to switch the magnetization from the +z to the −z direction by SOT with some value of the current. However, when the current is swept along [110] (red dots), we only observe magnetization reversal when the current flow and the bias are in the same direction. This behavior results in opposite chiralities for current scans along $\left[1\bar{1}0\right]$ and [110], as indicated by the black and red dashed arrows inside the hysteresis loops in Figure 7.

Three additional features seen in Figure 7 should be noted. First, we see that—independent of the current and bias arrangement, as well as the orientation of M—the value of AHR tends to decrease at the highest values of the current. This is the effect of σ increasing with increasing current, which tends to turn the magnetization M away from the easy axis and toward the xy-plane, thus reducing the value of AHR. Importantly, however, if it were not for the bias field, M would never switch signs, as has already been emphasized.

The second feature characteristic of these experiments is that the switching of M is not abrupt, but gradual, as manifested by the slope of AHR near the point of magnetization reversal. The reason for this is that the switching occurs as a multi-domain process, spread over a range of J, owing to differences of domain pinning at various points in the sample [29,30,31].

Finally, the chirality of the Hall resistance hysteresis observed as the current is swept back and forth is a feature of major importance. As an example, in Figure 7 this chirality is clockwise (CW) for current along [100], and counterclockwise (CCW) when current is scanned along $\left[1\bar{1}0\right]$. This is a clear indication that the Dresselhaus spin-orbit field is dominant, as can be seen by comparing Dresselhaus and Rashba spin-orbit fields in Figure 5b. If the Rashba field were dominant, the chirality would be the same for current scans in along both $\left[1\bar{1}0\right]$ and [110] directions.

#### 3.1.3. Quantification of Dresselhaus and Rashba Fields

**Figure 8:**
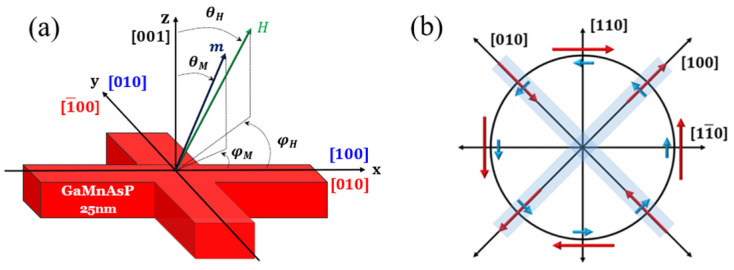
(**a**) Schematic diagram of the Hall bar device. The x direction is defined by the direction of positive current, which can flow either along [100] or [010]. (**b**) Directions of Rashba and Dresselhaus fields (blue and red arrows, respectively) for current directions indicated by black arrows. Note that when current flows either along [100] or [010], Dresselhaus and Rashba fields are orthogonal.

As has been pointed out, the chirality of the AHR hysteresis can tell us which of the two spin-orbit fields—Rashba or Dresselhaus—is dominant in a given situation. However, this does not determine their values. To quantify these two fields, it is convenient to use sample structures such as that shown in Figure 8, with current channels along the [100] and [010].

**Figure 9:**
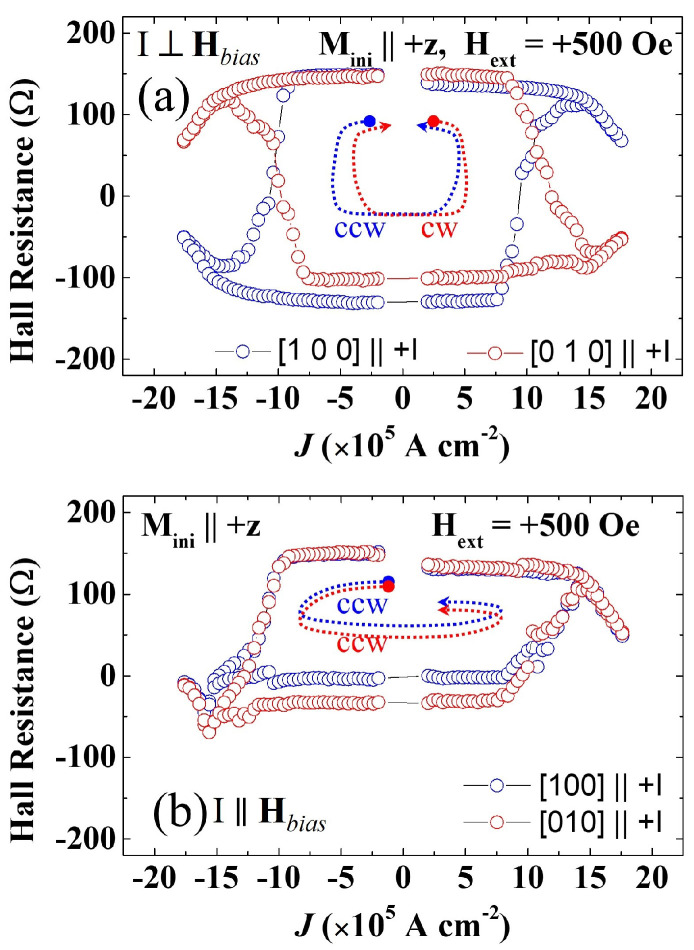
Hall resistance hysteresis loops measured on a (Ga,Mn)(As,P) film as a function of current at 55 K. Panel (**a**) shows AHR hysteresis for the $I⊥H_{\mathrm{bias}}$ configuration; (**b**) for $I∥H_{\mathrm{bias}}$ (**b**). Initial orientations of magnetization, current directions, and bias fields used in the measurements are shown in the panels. Hysteresis chiralities are indicated by dotted arrows. The starting points of each scan are marked by blue and red solid circles. Reprinted with permission from Ref. [28]. Copyright 2022, AIP Publishing.

Using this form of Hall device, HR measurements were performed at 55 K by scanning the current in the presence of an in-plane bias field $H_{\mathrm{bias}}$. The results for currents in the [100] and [010] directions are shown in Figure 9. The data shown in the top panel were obtained with the in-plane bias field perpendicular to the current, $I⊥H_{\mathrm{bias}}$, while the data in the bottom panel were taken when the bias and current are collinear, $I∥H_{\mathrm{bias}}$. Note that the chirality of SOT switching hysteresis loops observed for the [100] and [010] current scans (indicated by dotted arrows in Figure 9) is opposite for the two current directions when $I⊥H_{\mathrm{bias}}$, while it is the same when $I∥H_{\mathrm{bias}}$. This is because the specific arrangement of $H_{\mathrm{bias}}$ relative to I serves to “filter” either the effect of the Rashba field or the Dresselhaus field.

For example, consider the Dresselhaus field for the current flowing along [100]. As seen in Figure 8b, spin polarization σ produced by that field is parallel to the current, and when magnetization is rotated by σ to the layer plane (as discussed in Figure 6), it will require an $H_{\mathrm{bias}}$ perpendicular to σ (and thus to the current) in order to flip the magnetization form +z to −z directions, as illustrated in the upper panel of Figure 9. On the other hand, the Rashba field (and thus the spin polarization σ which it produces) is perpendicular to the current and tends to rotate the magnetization to the layer plane but perpendicular to the current. In this case, to rotate magnetization from +z to −z, one requires an $H_{\mathrm{bias}}$ that is parallel to the current. This is the case in the lower panel of the figure. Note that the chiralities of the two hystereses in the upper panel (which correspond to the effect of the Dresselhaus field) are opposite for [100] and [010] currents, while they are the same in the lower panel, as expected for the effect of the Rashba field. The fact that the amplitude of the HR hysteresis is larger for the $I⊥H_{\mathrm{bias}}\;$ configuration than for $I∥H_{\mathrm{bias}}$ is consistent with the fact that, as already noted, in the specimens used in this investigation, the Dresselhaus field is larger than the Rashba field.

The ability to separate the Dresselhaus and Rashba fields in this way provides the opportunity to quantify them [28]. This is accomplished by measuring the Hall resistance HR as an external magnetic field is rotated from the z axis either toward the current or in the plane perpendicular to the current while the current is held constant [28]. Note that such a rotation of the field effectively varies the field bias, either in the $I∥H_{\mathrm{bias}}$ or the $I⊥H_{\mathrm{bias}}\;$ configuration. Since the magnetization transition angle monitored by HR occurs at slightly different field angles for opposite current polarities due to the opposite direction of SOF generated by the current, the difference between these angles, Δθ, provides a measure of the effective SOF, as given by the relation $H_{eff}=H_{ex}\cdot \Delta \theta$ [16,26,32].

**Figure 10:**
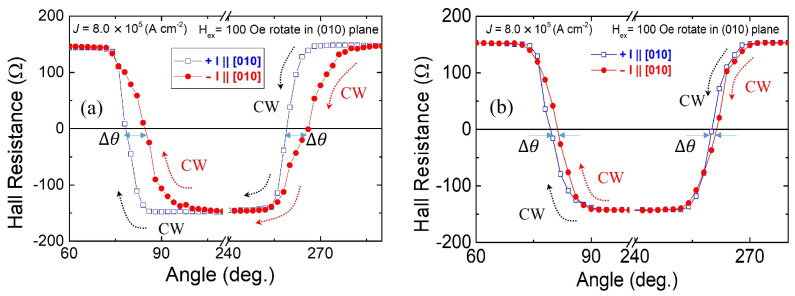
AHR measured as function of orientation of a rotating 100 Oe field at currents density of +8.0 × 10^5^ A/cm^2^ and −8.0 × 10^5^ A/cm^2^. (**a**) panel shows results for CW rotation of the field in the yz plane (i.e., the (010) crystal plane, perpendicular to the current). (**b**) panel shows data obtained with CW rotation of the field in the plane containing both the out-of-plane axis z and the current direction x (i.e., the (100) crystal plane). Dotted arrows indicate the directions of field rotation. The difference in switching angles Δθ between the sweeps with opposite current polarities provides the magnitudes of $H_{\mathrm{eff}}^{D}$ and $H_{\mathrm{eff}}^{R}$. Reprinted with permission from Ref. [28]. Copyright 2022, AIP Publishing.

The ***HR*** hysteresis loops obtained as a function of angle for opposite currents along the [010] direction are shown in Figure 10. Panel (a) shows data obtained by rotating a field of 100 Oe clockwise (CW) in the yz-plane (i.e., the (010) crystal plane), i.e., the plane which is perpendicular to the Dresselhaus SOF for the [010] current direction, while panel (b) shows data observed by rotating the field in the xz-plane (i.e., the (100) plane), i.e., the plane which perpendicular the Rashba SOF for the same current direction. Thus, the value of Δθ in Figure 10a provides the Dresselhaus effective SOF, $H_{eff}^{D}$, and 10b provides the value of the Rashba SOF, $H_{eff}^{R}$.

The Dresselhaus and Rashba SOFs at a current density of 8.0 × 10^5^ A/cm^2^ obtained from these data are $H_{eff}^{D}=7.5\;\mathrm{Oe}\;$ and $H_{eff}^{R}=2.0\;\mathrm{Oe}$, respectively. The $H_{eff}^{D}$ is about four times larger than $H_{eff}^{R}$, consistent with other studies on GaAs-based ferromagnetic semiconductor films with out-of-plane anisotropy [26,31]. One should note, however, that the specific values of these fields can vary from sample to sample, since they depend on strain as well as on the material with which a given sample is interfaced.

#### 3.1.4. Really Field-Free SOT Switching

**Figure 11:**
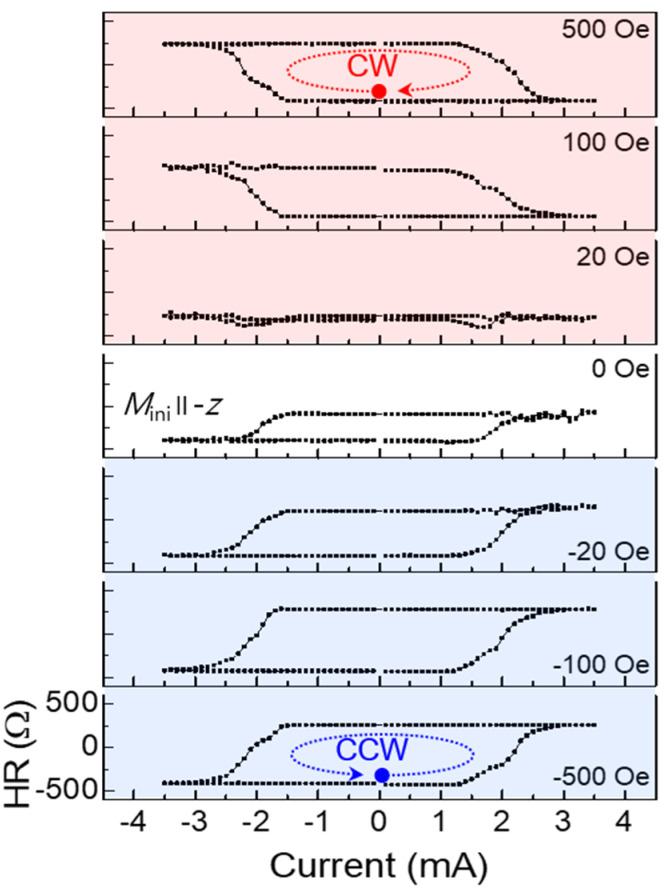
Hall resistance hysteresis loops as a function of current measured at 2.5 K under several values of in-plane bias fields applied along the current. Shaded regions show different configurations of initial magnetization and in-plane bias (i.e., different combinations of $M_{i}∥\;\pm \;z$ and $H_{\mathrm{bias}}∥\;\pm \;x$). The chirality of SOT hysteresis loops is indicated by dotted arrows inside the loops. The starting point of the scan is indicated by the solid dot. Note that the hysteresis vanishes at *H*_bias_ ≈ 20 Oe, not at $H_{\mathrm{bias}}=0$. Adapted from Ref. [33].

In recent studies on SOT switching, we—quite unexpectedly—observed that, contrary to the arguments stated earlier, we did not need to apply an external field bias to achieve field reversal of magnetization perpendicular to the sample plane [33]. We explain this result by assuming that there is an oxidation of Mn on the sample surface, forming a magnetic but as yet unidentified Mn oxide. We assume that this surface oxide becomes magnetized as the initiating field is applied and provides a magnetic bias that then results in a rotation of the magnetization, similar to the effect of magnetic bias used normally in SOT magnetization reversal. The results obtained as a function of applied field bias in Figure 11 support this argument, showing that even when the applied bias vanishes, the observed AHR displays a hysteresis, requiring the application of a bias in the opposite direction for the hysteresis to vanish. While at this moment, we do not have a full explanation of the observed effect, and we cannot provide a definitive description of the structure of the Mn oxide, the fact that such systems can be grown is in itself a most encouraging result, showing promise that—once the nature of the oxide layer has been identified—FMS samples not requiring an external bias can be prepared and used to manipulate magnetization by SOT.

#### 3.1.5. Efficiency of Spin-Orbit Torque

SOT efficiency is defined as effective spin-orbit field per unit current density *J*, *χ* ≡ *H*_SO_*/J*. In our case, the highest efficiency (7.4 Oe/10^5^ A × cm^−2^) was observed when the current was flowing along the $\left[1\bar{1}0\right]$ direction of the crystal, for which Dresselhaus and Rashba SOFs were parallel and of the same sign in the film [31]. In HM/FM systems, the efficiency is typically one to two orders of magnitude lower than this value [32,34,35], indicating the suitability of FMS layers for these applications.

**Figure 12:**
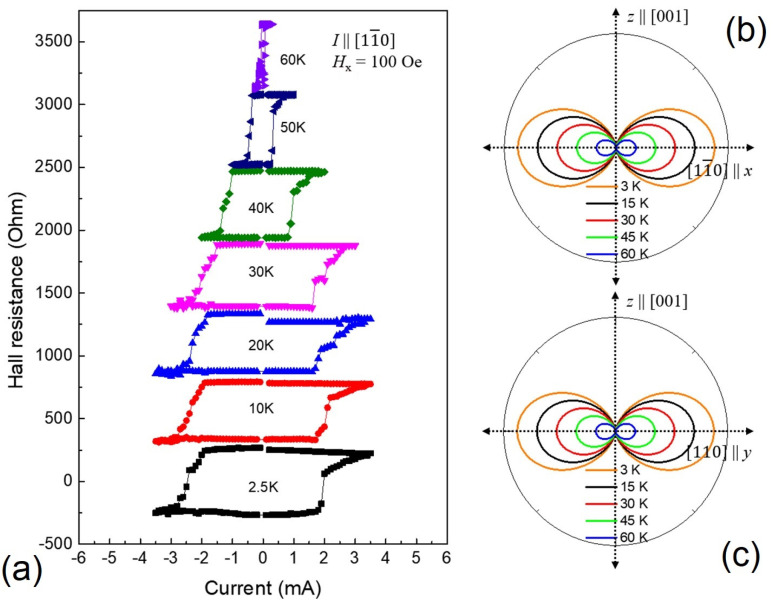
(**a**) HR hysteresis loops as a function of current scanned along the $\left[1\bar{1}0\right]$ direction, with H*_bias_* = 100 Oe at several temperatures. Hysteresis loops are shifted upward for clarity. The switching chirality of the hysteresis loops is CCW owing to the $M_{i}∥+z$, $H_{\mathrm{bias}}∥+I$ configuration. Note that the current density required for reversing magnetization decreases rapidly as temperature is increased since, as shown on the right, (**b**,**c**) the energy barrier opposing magnetization reversal decreases with increasing temperature. Reprinted with permission from Ref. [36]. Copyright 2024, IEEE.

Here, one should also discuss the current itself needed to flip magnetization. Importantly, as the temperature increases, the energy barrier to be overcome for switching magnetization systematically decreases, thus requiring lower SO fields (and thus lower currents) to reorient the magnetization at higher temperatures (see Figure 12). For example, in [36], we showed that, as the temperature increased from 2.5 K to 65 K, the critical current needed for switching magnetization systematically decreased from J_c_~18 × 10^5^ A/cm^2^ at 2.5 K to J_c_ ≈ 4.0 × 10^3^ A/cm^2^ at 65 K [36].

### 3.2. In-Plane Magnetization Switching

So far, we have discussed out-of-plane manipulation of M, which is of primary importance in layers whose magnetization is naturally oriented normal to the layer plane. When the dominant easy axes lie in the plane of the layer, we will be interested in manipulating in-plane M. In this case, as can be seen from Equation (3), the behavior of in-plane magnetization can be studied experimentally by measuring the planar Hall resistance, given by

(5) $$HR=\frac{k}{t}M_{∥}^{2}\sin2\varphi_{M}.$$

We note parenthetically that planar Hall resistance (PHR) is not so much a Hall resistance as it is a manifestation of anisotropic in-plane magnetoresistance.

The plane of the ferromagnetic semiconductor of the (Ga,Mn)As family is magnetically anisotropic, and current carriers with different spins scatter differently to the left and to the right, resulting in a Hall-like voltage that—as seen in Equation (5)—is proportional to *M*^2^. The origins of this effect are currently still a matter of debate, with various models that involve skew scattering, side-jump scattering, and/or the effects of the Berry phase [37]. Empirically, however, its behavior is well described by Equation (5), thus providing a convenient tool for measuring magnetization when it lies in the plane of the sample.

#### 3.2.1. SOT Magnetization Switching in the Plane of the FMS Film

**Figure 13:**
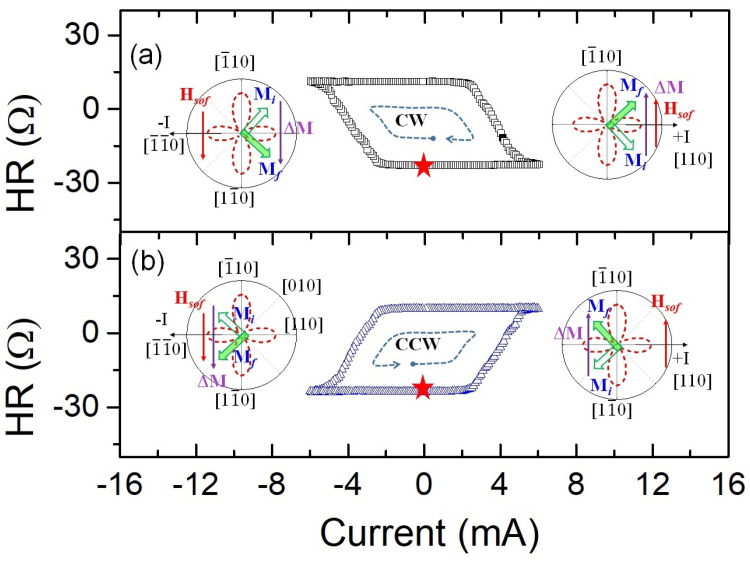
HR hysteresis loops obtained at 2.5 K by scanning the current along the [110] direction. No external magnetic field is applied. Panel (**a**) shows HR data for initial magnetizations either along the 45° or 315° easy axes; in panel (**b**) initial magnetizations were at either 135° or 225°. The insets in each panel show the alignment of spin-orbit fields $H_{\mathrm{sof}}$ and the corresponding magnetization ΔM change for SOT switching obtained with negative and positive currents. Black, purple, and red arrows represent directions of current ***J***, ΔM, and $H_{\mathrm{sof}}$, respectively. Open and solid green arrows in the insets show directions of magnetization before ($M_{i}$) and after ($M_{f}$) the transition. The initial point for each current scan is marked by the star. Dotted arrows in hysteresis loop indicate the SOT switching chirality. Adapted from Ref. [38].

In this set of experiments, we will deal with FMS layers under compressive strain, where the easy axes are in the layer plane, as shown on the left of Figure 4. Typical SOT magnetization switching in a (Ga,Mn)As film is shown in Figure 13, where HR data for current scans along the [110] direction (which we define as positive current direction) and along $\left[\bar{1}\bar{1}0\right]$ (i.e., negative current direction) are obtained in the absence of an external field. The observed field-free in-plane SOT switching in a (Ga,Mn)As film is possible because the net SOF (i.e., the vector sum of Dresselhaus- and Rashba-type SOFs shown in Figure 3) has a parallel component to the direction of magnetization change, i.e., $\Delta M=M_{f}-M_{i}$, where $M_{i}$ and $M_{f}$ are magnetizations before and after the transition, respectively [38]. For example, magnetization in the [010] direction (see green open arrow at 45° shown in the left inset of Figure 13a) can be switched by the application of a negative current, which generates SOF along the $\left[1\bar{1}0\right]$ direction (see the red arrow in left inset of Figure 13a). This SOF is parallel to the vector ΔM for magnetization transition from the [010] direction to [100] (see the violet arrow in left inset of Figure 13a). This SOF causes a CW rotation of magnetization over the [110] barrier when the current in the negative direction reaches a critical value. The same switching process occurs during a positive current scan when the initial magnetization is in the [100] direction, shown in the right inset of Figure 13a. This switching process results in the 90° field-free SOT magnetization switching between the [010] and [100] directions over the [110] barrier during the current scan, when the magnetization is initially aligned along the [010] direction. A similar switching process occurs when the magnetization is initialized along the [100] direction, as shown in Figure 13b. We emphasize that, unlike the case of magnetization switching perpendicular to the plane of the film, transitions between magnetization states in the in-plane switching process do not require any external magnetic bias—a property that may be desirable in certain device applications.

#### 3.2.2. Quantification of Dresselhaus and Rashba Fields in In-Plane Film

As in quantifying spin-orbit fields for out-of-plane magnetization switching, one can also use current channels along the [100] and [010] to quantify the Dresselhaus and Rashba fields in in-plane magnetization switching, again because the two types of fields are orthogonal when the current flows along these directions. However, unlike out-of-plane studies, in this case, it is convenient to use anisotropic magnetoresistance (AMR) rather than PHR. In crystalline (Ga,Mn)As films, AMR shows a $\sim \cos2\varphi_{M}$ dependence, where $\varphi_{M}$ is the orientation of magnetization in the layer plane [39,40,41,42], which we can use to monitor the magnetization of the film and, when current is flowing, to obtain the values of SOFs induced by the current, as discussed below.

**Figure 14:**
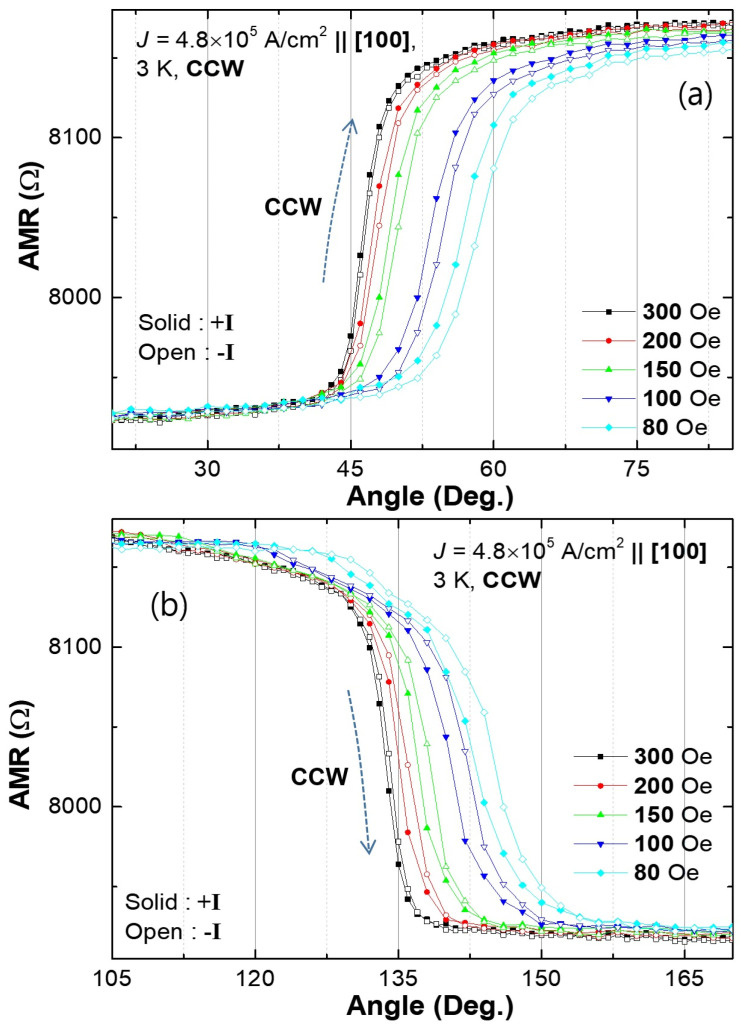
Anisotropic magnetoresistance as a function of angle of a constant magnetic field rotated CCW near the [110] barrier ($\varphi_{H}=45{}^{\circ}$, (**a**)) and the $\left[\bar{1}10\right]$ barrier (135°, (**b**)) measured at 3 K when current density of 4.8 × 10^5^ A/cm^2^ flows along the [100] direction. Solid and open symbols represent data obtained with positive and negative currents, respectively. Note that the splitting of transition angles between positive and negative currents increases as the magnitude of the rotating field decreases. Adapted from Ref. [43].

Han et al. [43] showed that in experiments involving a constant magnetic field rotated in the layer plane, the values of the Dresselhaus (H_D_) and Rashba (H_D_) fields can be obtained from the difference in the angle at which magnetization transition occurs over the [110] and $\left[\bar{1}10\right]$ barriers for opposite current polarities. As an example, Figure 14 shows the magnetization transitions over the barriers at φ= 45° and φ=135° for the CCW field rotation. The transitions shift to higher angles for smaller rotating field values, as would be expected, but display a hysteresis between opposite current directions. As described in [43], the splitting of transition angles between the two current polarities can then be analyzed to provide the values of the $H_{D}$ and $H_{R}$ separately. The values of SOFs obtained in [43] are *H_D_* = 1.69 ± 0.08 Oe and *H_R_* = 0.14 ± 0.08 for a current density of $4.8\times 10^{5}\;A/{\mathrm{cm}}^{2}$. As in out-of-plane experiments, here also, Dresselhaus-type SOFs larger than Rashba-type fields were consistently observed on (Ga,Mn)As and related films [44,45,46].

#### 3.2.3. Manipulation of In-Plane Magnetization States by Alternating Current Pulses

**Figure 15:**
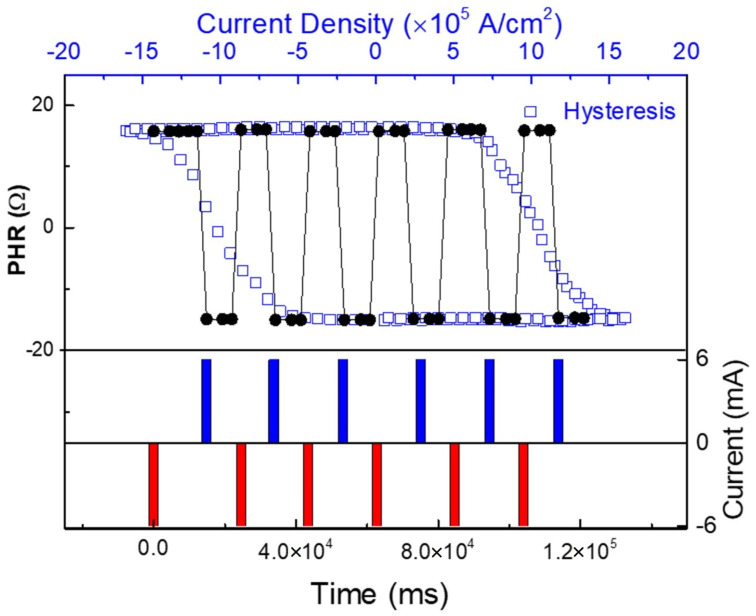
Time dependence of PHR observed after each 6 mA current pulse, showing field-free SOT magnetization switching as current polarity is changed. The PHR values obtained using the alternate 6 mA current pulses remain unchanged until the next pulse is applied, and match perfectly the PHR values of the hysteresis obtained by scanning the current (blue squares). Adapted from Ref. [38].

For spintronic device applications, manipulation of magnetization without an external field is of a crucial importance. In Figure 15, we show an example of field-free SOT switching by using alternate current pulses of opposite polarity in a (Ga,Mn)As film with in-plane magnetic easy axes. For reference, we also plot the hysteresis obtained by scanning the current in both directions (open squares), similar to Figure 13, showing two magnetization states that correspond to the two orientations of M. In this experiment, the magnetization is first initialized along the [010] direction, and a sequence of 6 mA pulses is then applied alternately in opposite directions, with each pulse of 10 ms duration. After each pulse, PHR resulting from the pulse is measured with a small direct current of 20 μA, plotted as solid circles in Figure 15. As the pulse polarity is reversed, the PHR value reverses accordingly as magnetization is switched from one easy-axis orientation to the other by the SOF generated by the current pulse, as plotted with blue open squares. Importantly, each state remains at a constant value until the next current pulse with the opposite polarity is applied. This clearly demonstrates that the SOT generated by the current pulses switches the magnetization of the (Ga,Mn)As film between the two orthogonal in-plane easy axes without the need of an applied magnetic field. The stability of the states in the device further suggests the potential of field-free SOT memory applications.

## 4. Summary and Future Directions

In this review, we have shown that, to manipulate magnetization by an electric current, we need the current to be spin-polarized. To achieve such spin-polarization, the material is required to have broken inversion symmetry. Inversion asymmetry in materials of interest in this review can arise either from intrinsic properties of the crystal (which then gives rise to the Dresselhaus effect), or from its macroscopic layer structure (which leads to the Rashba effect). Both effects are relativistic, arising from Lorentz transformation of the internal electric field which, when transformed to the frame of reference of current carriers, results in a magnetic field that polarizes the spin of the carriers.

Measurements of magnetization reversal may be conveniently monitored by Hall resistance. In this context, we have made special note of the chirality of the Hall-resistance hysteresis, which is different for the Rashba and the Dresselhaus effect, thus offering a means for distinguishing between the two processes. We have also noted that the process of magnetization switching from one easy axis to another occurs domain-by-domain. In the specific case of magnetization switching from one out-of-plane orientation to the opposite orientation, we have also noted that this process requires a magnetic-field bias. Such bias field can be externally applied, or—as indicated in our most recent experiments—it can result from an internal magnetization in an adjacent film, such as an (as yet unidentified) Mn oxide. In contrast, in-plane switching of magnetization (by 90°) occurs completely field-free.

We have devoted considerable attention to a quantitative discussion of the efficiency of manipulating magnetization by SOT in single FMS films, and we showed that the switching efficiency in FMS single films is considerably more efficient than in HM/FM bilayers. FMS films offer distinct advantages over FM/HM bilayer systems for SOT switching by their inherent strong spin-orbit interaction and high spin polarization, and by eliminating the need of an interface between the polarized spin source and the FM layer. We note finally that, since magnetization switching depends on magnetic “hardness” of the material, in the case of FMS systems switching of magnetization at higher temperatures requires significantly lower currents, which may be advantageous in some situations.

Despite the interesting SOT phenomena occurring in crystalline FMS materials and their advantages for manipulating magnetization by electric current, many challenges still remain for achieving SOT-based applications. To make further advances in this field, several critical issues need to be addressed:

1. While the fundamental mechanisms of SOT are generally understood, there is a notable lack of systematic quantitative analysis regarding the contributions from FLT and DLT across various materials systems. In particular, one needs to determine the precise magnitudes of these contributions, and one needs to establish how they influence the dynamics of magnetization. In this regard, simulations based on the LLG equation and the relative strengths of FLT and DLT components have already highlighted the critical role of the DLT process in the switching behavior in FMS films [17,47]. Furthermore, simulation studies of SOT switching in the presence of an in-plane external field have emphasized the importance of relative alignments between the torque arising from an external field and the current-induced field-like torque (FLT), which can impede the SOT switching process [17,47]. Future experimental investigation is needed to address the quantitative aspects of FLT and DLT in FMS films, and to adapt specific techniques, such as second-harmonic measurements, which can quantify FLT and DLT contributions separately [48].
2. Recognizing that magnetization switching occurs domain-by-domain, a deeper understanding of electrical control of magnetic domain walls is essential, particularly regarding their movement and behavior in the presence of disorder and Dzyaloshinskii-Moriya interactions (DMI) [31]. Clearly domain wall nucleation and propagation are critical to SOT-driven switching. To fully understand SOT switching in crystalline FMSs, numerical simulations using first-principles rules to compute the dynamic evolution of micromagnetic systems are especially important. However, such simulations often face challenges due to their slow computational speed resulting from the need for global convolution for calculating demagnetizing fields and DMI, which require taking into account comprehensive interactions among all units in the specimen. Additionally, limited knowledge about the domain texture and domain wall configuration in FMS films [49], as well as their relations with disorder, complicates these simulations further. Progress in this area will constitute an important step in the development of advanced SOT-based spintronic devices.
3. An issue of major importance in developing practical spintronic devices is to achieve field-free (FF) operation of the device. Importantly, SOT-induced magnetization switching without an external magnetic field has already been observed in FMS films exhibiting both in-plane and out-of-plane magnetic anisotropy in certain situations [16,23,38]. Although a qualitative link between such field-free switching and spin polarization aligned with an internal magnetic field has already been established, a comprehensive understanding of FF-SOT switching is still lacking. In addition, it is also necessary to find new ways of achieving FF-SOT switching in FMS films. As already demonstrated by several preliminary studies, this can be achieved by creating broken inversion symmetry in crystalline FMS films by introducing a strain gradient [20,21,30,50], by tilting magnetic anisotropy in the film [25], or by generating an out-of-plane component of spin polarization.
4. Even though the SOT switching efficiency in FMS single films is already significantly better than in HM/FM bilayers, it can be further improved via strategic design of structures, involving additional layers that themselves have spin polarization. For example, the surface states of topological insulators (TIs) are protected by time-reversal symmetry and exhibit a Dirac-like linear dispersion characterized by spin-momentum locking. This property makes them particularly attractive for SOT and other spintronic applications. While crystalline FMSs are typically grown on (001) substrates, high-quality hexagonal TIs have been successfully grown on (001) GaAs substrates [51]. This advancement allows for the study of SOT in bilayer systems such as Bi₂Se₃/GaMnAsP. The exotic spin texture of the Dirac cone holds the potential of enhancing SOT efficiency [52]. Specifically, a current flowing through the topological surface states can generate a nonequilibrium spin density with both in-plane and out-of-plane components, thereby inducing torques—both out-of-plane and in-plane—on an adjacent magnetic layer. It is worth noting that crystals exhibiting a giant Rashba effect, such as BiTeI [53] and GeTe [54], as well as various low-dimensional systems [55,56], hold great promise for significant charge-spin conversion and enhanced SOT efficiency when interfaced with FMS layers.
5. Finally, search for the most efficient sources of SOT prompts inquiries into the nature of SOT itself, especially in systems with very large spin-orbit coupling and novel spin textures. In this context, it is important to highlight that other FMS systems exhibiting bulk inversion asymmetry (BIA) also merit exploration for SOT applications. Promising candidates include zinc-blende (Ga,Mn)(As,Bi) [57], (In,Fe)As [58] and (Ga,Fe)Sb [59], wurtzite (Ga,Mn)N [60], and rhombohedral (Ge,Mn)Te [61]. Despite their potential, these materials have received relatively little attention to date. One reason for this may be our limited understanding of transport mechanisms in these systems, particularly regarding spin-charge conversion. A deeper investigation into these materials is likely to unveil new pathways for efficient SOT processes, thus significantly advancing the field of spintronics.

## Figures and Tables

**Figure A1:**
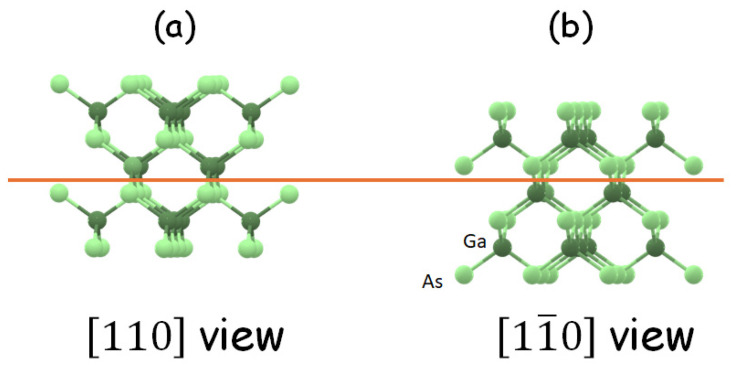
Zinc blende crystal structure of GaAs showing views along (nearly) [110] and $\left[1\bar{1}0\right]$ crystallographic directions. A charge carrier moving along [110] “sees” an internal vertical electric field that is opposite to that which it “sees” when moving along $\left[1\bar{1}0\right]$.

## Data Availability

Not applicable.

## Appendix A

The Rashba and Dresselhaus effects are both relativistic, resulting from transforming the electric field that exists in the system into magnetic field in the frame of reference of the moving carrier, which then results in spin polarization of the carrier. We recall that the Lorentz transformation is given by $B=-\gamma \left(v\times E\right)/c^{2}$. In the case of the Rashba effect it is easy to visualize the process because, as we discussed, in that case E results from the discontinuity of the potential and is naturally perpendicular to the layer plane, while v is in the plane of the layer. This results in a magnetic field B that has the same chirality, as shown in Figure 3, regardless of the crystallographic direction in which the current flows.

In the case of the Dresselhaus effect, it is more difficult to visualize the internal electric field, since it naturally depends on the crystalline direction. For example, when the current carrier is moving along the [110] direction, it “sees” the atomic distribution as shown in Figure A1a, but when the current flows along $\left[1\bar{1}0\right]$, the atomic configuration “seen” by the carrier is given by Figure A1b. Since the internal electric field results from electronegativity of the two elements (e.g., Ga and As), one can thus conclude that the signs of E are opposite in the two cases, and thus the chirality of B with respect to v is also opposite, as shown in Figure 3. This is the underlying reason why the spin polarizations arising from the Dresselhaus effects are opposite for currents flowing along [110] and $\left[1\bar{1}0\right]$ directions, as seen in Figure 3.

In Figure 3 we also see that when current flows along either [100] or [010], the Dresselhaus field is along the current (i.e., along v), which may at first be puzzling in the context of Lorentz transformation. This arises, however, because to obtain the Dresselhaus spin-orbit field along the current, we must resolve ***v***_[**100**]_ into its components along [110] and $\left[1\bar{1}0\right]$, obtain B for each of these components, and add the result vectorially. We then obtain the dependence of the Dresselhaus field exactly as shown in Figure 3.
